# Supplementary material for: Multi‐dataset identification of innovative feature genes and molecular mechanisms in keratoconus
Source: J Cell Mol Med. 2024 Sep 19;28(18):e70079. doi: 10.1111/jcmm.70079 (PMC11412914; doi:10.1111/jcmm.70079)
Supplement: Supplementary file 2 — Material S2. [file JCMM-28-e70079-s002.zip › 7.GSEA analysis/3.DBNDD1/KEGG_RNA_DEGRADATION.html]

Details for gene set KEGG\_RNA\_DEGRADATION[GSEA]

|  || Dataset | DBNDD1.group.cls#h\_versus\_l |
| Phenotype | group.cls#h\_versus\_l |
| Upregulated in class | h |
| GeneSet | KEGG\_RNA\_DEGRADATION |
| Enrichment Score (ES) | 0.260338 |
| Normalized Enrichment Score (NES) | 1.3215976 |
| Nominal p-value | 0.047524754 |
| FDR q-value | 0.66477555 |
| FWER p-Value | 0.943 |
Table: GSEA Results Summary

  

Fig 1: Enrichment plot: KEGG\_RNA\_DEGRADATION      
 Profile of the Running ES Score & Positions of GeneSet Members on the Rank Ordered List

  

| SYMBOL | TITLE | RANK IN GENE LIST | RANK METRIC SCORE | RUNNING ES | CORE ENRICHMENT || 1 | EXOSC6 | na | 7222 | 0.119 | -0.1417 | Yes |
| 2 | PATL1 | na | 8466 | 0.095 | -0.1244 | Yes |
| 3 | DDX6 | na | 8615 | 0.092 | -0.0772 | Yes |
| 4 | CNOT6 | na | 9378 | 0.080 | -0.0545 | Yes |
| 5 | DCP2 | na | 9632 | 0.076 | -0.0192 | Yes |
| 6 | CNOT6L | na | 10662 | 0.062 | -0.0140 | Yes |
| 7 | LSM4 | na | 11878 | 0.048 | -0.0222 | Yes |
| 8 | CNOT1 | na | 11983 | 0.047 | 0.0011 | Yes |
| 9 | DCP1B | na | 12291 | 0.044 | 0.0170 | Yes |
| 10 | DCPS | na | 12299 | 0.044 | 0.0415 | Yes |
| 11 | ZCCHC7 | na | 12797 | 0.040 | 0.0494 | Yes |
| 12 | EXOSC3 | na | 13837 | 0.031 | 0.0369 | Yes |
| 13 | EDC3 | na | 14091 | 0.029 | 0.0460 | Yes |
| 14 | PAPOLB | na | 14149 | 0.029 | 0.0604 | Yes |
| 15 | LSM3 | na | 14740 | 0.025 | 0.0571 | Yes |
| 16 | ENO1 | na | 14910 | 0.023 | 0.0654 | Yes |
| 17 | LSM1 | na | 15996 | 0.016 | 0.0429 | Yes |
| 18 | EXOSC1 | na | 16284 | 0.014 | 0.0425 | Yes |
| 19 | EXOSC4 | na | 16368 | 0.013 | 0.0475 | Yes |
| 20 | LSM7 | na | 16856 | 0.010 | 0.0392 | Yes |
| 21 | EXOSC2 | na | 17299 | 0.008 | 0.0307 | Yes |
| 22 | LSM2 | na | 17720 | 0.005 | 0.0213 | Yes |
| 23 | CNOT10 | na | 17967 | 0.003 | 0.0158 | Yes |
| 24 | XRN1 | na | 18027 | 0.002 | 0.0155 | Yes |
| 25 | CNOT3 | na | 18084 | 0.002 | 0.0150 | Yes |
| 26 | PAPOLA | na | 18419 | -0.000 | 0.0055 | Yes |
| 27 | EXOSC5 | na | 18692 | -0.002 | -0.0011 | Yes |
| 28 | LSM5 | na | 18770 | -0.003 | -0.0018 | Yes |
| 29 | LSM8 | na | 19349 | -0.007 | -0.0143 | Yes |
| 30 | CNOT7 | na | 19567 | -0.009 | -0.0155 | Yes |
| 31 | PNPT1 | na | 19716 | -0.011 | -0.0139 | Yes |
| 32 | HSPD1 | na | 19807 | -0.011 | -0.0102 | Yes |
| 33 | CNOT2 | na | 20319 | -0.016 | -0.0161 | Yes |
| 34 | EXOSC9 | na | 20480 | -0.017 | -0.0112 | Yes |
| 35 | PARN | na | 20735 | -0.020 | -0.0076 | Yes |
| 36 | LSM6 | na | 21259 | -0.024 | -0.0091 | Yes |
| 37 | C1D | na | 21446 | -0.026 | 0.0002 | Yes |
| 38 | EXOSC8 | na | 21536 | -0.027 | 0.0128 | Yes |
| 39 | EXOSC7 | na | 21606 | -0.028 | 0.0264 | Yes |
| 40 | DCP1A | na | 22030 | -0.032 | 0.0322 | Yes |
| 41 | ENO2 | na | 22371 | -0.036 | 0.0424 | Yes |
| 42 | DIS3 | na | 22700 | -0.040 | 0.0551 | Yes |
| 43 | HSPA9 | na | 22767 | -0.040 | 0.0758 | Yes |
| 44 | PAPOLG | na | 22821 | -0.041 | 0.0971 | Yes |
| 45 | EDC4 | na | 23108 | -0.044 | 0.1136 | Yes |
| 46 | XRN2 | na | 23313 | -0.047 | 0.1339 | Yes |
| 47 | CNOT8 | na | 23833 | -0.054 | 0.1491 | Yes |
| 48 | EXOSC10 | na | 24823 | -0.068 | 0.1585 | Yes |
| 49 | ENO3 | na | 24958 | -0.070 | 0.1937 | Yes |
| 50 | MPHOSPH6 | na | 25346 | -0.076 | 0.2249 | Yes |
| 51 | CNOT4 | na | 25700 | -0.082 | 0.2603 | Yes |
Table: GSEA details [plain text format]

  

Fig 2: KEGG\_RNA\_DEGRADATION      
 Blue-Pink O' Gram in the Space of the Analyzed GeneSet

  

Fig 3: KEGG\_RNA\_DEGRADATION: Random ES distribution      
 Gene set null distribution of ES for **KEGG\_RNA\_DEGRADATION**
